# Factors Associated with Nutritional Status in Grassroots Recyclers in Ecuador: A Machine Learning Approach

**DOI:** 10.3390/ijerph23020240

**Published:** 2026-02-14

**Authors:** Jenny Albarracín-Méndez, Diana Morales-Avilez, Francisco Arias-Pallaroso, Gabriele Davide Bigoni-Ordoñez, Andrea Gómez-Ayora

**Affiliations:** 1 Departamento Interdisciplinario de Espacio y Población (DIEP), Universidad de Cuenca, Cuenca 010107, Ecuador; diana.moralesa@ucuenca.edu.ec (D.M.-A.); alexander.arias@ucuenca.edu.ec (F.A.-P.); andrea.gomeza@ucuenca.edu.ec (A.G.-A.); 2 Carrera de Laboratorio, Facultad de Ciencias Médicas, Universidad de Cuenca, Cuenca 010107, Ecuador; gabriele.bigonio@ucuenca.edu.ec

**Keywords:** nutritional status, waste pickers, sociodemographic factors, working conditions, clinical laboratory test, machine learning, Ecuador

## Abstract

**Highlights:**

**Public health relevance—How does this work relate to a public health issue?**

- Grassroots recyclers are a vulnerable occupational group who are exposed to adverse environmental conditions, food insecurity, nutritional risks, social inequity and health inequity, constituting relevant public health concerns.
- This study links nutritional status with sociodemographic, health, and work-related factors, which are determinants of health in an under-researched informal working population in Ecuador.

**Public health significance—Why is this work of significance to public health?**

- The study provides empirical evidence on factors associated with nutritional status among grassroots recyclers, helping to address knowledge gaps related to health and nutrition in a vulnerable group.
- The application of machine learning models allows the identification of complex relationships among sociodemographic, territorial, occupational, and health-related variables relevant to public health research.

**Public health implications—What are the key implications or messages for practitioners, policy makers and/or researchers in public health?**

- These results support and strengthen, with empirical evidence, the design of public policies and targeted interventions, strategies that incorporate periodic nutrition assessment and monitoring of the grassroots recyclers.
- By integrating approaches grounded in equity, territory, the life course and occupational health approaches, these strategies provide actionable guidance for public health practitioners, policymakers and studies to reduce nutritional inequities and improve quality of life in this vulnerable population.

**Abstract:**

Grassroots recyclers play a fundamental role in solid waste management in Ecuador; however, they often work under precarious conditions that may compromise their health. This study aimed to identify factors associated with nutritional status, operationalized as the presence or absence of nutritional alterations, among grassroots recyclers through supervised machine learning approaches. Data from 303 recyclers from three Ecuadorian cities (Cuenca, Macas, and La Libertad) were analyzed, incorporating sociodemographic, occupational, and health-related variables. Nutritional alterations were defined based on anthropometric and biochemical indicators, specifically, excess body weight and/or elevated total lipid levels. The results showed that 71% presented nutritional alterations, evidencing an important public health problem in this vulnerable population. Significant associations were observed with sex, age, canton of residence, ability to ride a bicycle, bicycle use for work, and attendance at medical check-ups. Among the evaluated models, CatBoost trained with SMOTE achieved the highest ROC-AUC value and the most balanced performance between classes, although sensitivity for individuals without nutritional alterations remained limited. Feature importance analysis highlighted sociodemographic, occupational, economic, and healthcare access factors, underscoring the multidimensional nature of nutritional risk and supporting the use of machine learning as a support tool for public health planning and targeted interventions.

## 1. Introduction

Waste management in Ecuador relies heavily on grassroots recyclers, who work under informal conditions, earning low incomes and facing exclusion. Although their work is essential for society and the environment, they have historically been invisible in public health policies [1]. Assessing their nutritional status is essential as a key indicator of health.

Nutritional status is assessed through four complementary methods: anthropometric, biochemical, clinical, and dietary, commonly referred to as the ABCD of nutritional assessment. Anthropometric parameters, such as weight and height, are used to derive indicators like the Body Mass Index (BMI), while biochemical assessment involves the analysis of biological samples such as blood, urine, and stool. Clinical evaluation includes physical examination, and dietary assessment is conducted through dietary surveys. These methods provide indicators that reflect different aspects of nutritional status; however, no single indicator offers a complete evaluation. Therefore, their combined use is recommended to obtain a comprehensive and accurate assessment of an individual’s nutritional status [2]. BMI allows people’s weight to be classified as normal weight, overweight, or obese [3]. The latter two conditions are considered forms of malnutrition due to excess and constitute global health problems that affect different social strata.

In Ecuador, the lack of studies on this issue among waste pickers creates a significant gap in understanding the associated factors, limiting the ability to develop interventions tailored to their needs. This has a direct impact on health, leading to the development of chronic diseases, including diabetes, hypertension, dyslipidemia, cardiovascular disease, osteoarthritis, and certain types of cancer [4].

Globally, overweight and obesity have reached epidemic proportions. According to the World Obesity Atlas 2024 report, it is projected that by 2035, 54% of the world’s population will be affected by excess weight. The most significant increases will be in low- and middle-income countries [5]. This trend is related to multiple structural determinants, including food insecurity and the low availability of nutritious foods, which lead to poor and unhealthy eating habits, low nutritional quality, and high caloric density [6].

Previous studies have shown that malnutrition due to excess is associated with sociodemographic, economic, and occupational factors. Obesity is inversely associated with education [7] and socioeconomic status [8]. People from low socioeconomic strata, racial and ethnic minority groups, older adults, and women face greater barriers to accessing a varied diet, which influences the development of excess weight [9,10,11,12]. In the case of women, this prevalence is associated with economic constraints, constant exposure to food insecurity, and traditional gender roles [10]. In addition, age is positively associated with BMI, due to lower physical activity and less healthy eating habits [13]. Marital status and occupation also have an impact, with high BMI being more common in married people or those on sick leave or disability [13].

In contexts of structural exclusion, such as those faced by grassroots recyclers, nutritional status can be affected by multiple factors. This population has low educational levels [6,14,15], limited economic resources [12,16,17], and a high dependence on recycling as a means of subsistence [15]. In addition, they have long working hours [16], lack basic services [6], and are constantly exposed to occupational hazards [6,16,17,18,19]. The impact of these conditions may vary depending on factors such as gender, age, ethnicity, or employment status.

Previous studies show a high prevalence of overweight and obesity in this population [12,20], as well as respiratory diseases, tuberculosis, and metabolic disorders such as dyslipidemia, hypertension, and diabetes, linked to poor access to health services [6]. Although excess weight is often associated with cardiovascular disease, some studies indicate that this relationship is not always significant. However, it is present in metabolic factors such as hypertension, hyperglycemia, hypercholesterolemia, and hypertriglyceridemia [20]. An association has also been identified between high triglycerides, excess weight, and insulin resistance, reinforcing the link between metabolic disorders and biochemical alterations [21].

Other studies have shown that the prevalence of overweight and obesity among workers engaged in solid waste collection is linked to the consumption of foods with high energy density and low nutritional quality. Lack of access to health services and inadequate food education also act as structural determinants of malnutrition in this group [15]. In addition, economic insecurity has been identified as a central factor in the emergence of overweight and obesity among waste pickers [12].

Despite international evidence, there is still a lack of comprehensive studies in Ecuador on the nutritional status of waste pickers and their relationship with sociodemographic, occupational, and health variables. This gap hinders the design of effective public policies and interventions, which in turn limit improvements in the quality of life of this population group.

In response to this, the present study aims to identify the determinants of the nutritional status of waste pickers in three Ecuadorian cities, as well as to characterize the profiles of those with adequate nutritional status and those without. It is hoped that these results will generate sound evidence for the formulation of public policies and programs aimed at improving the overall health of this population.

The relevance of this study lies in its ability to highlight the health conditions of a vulnerable and marginalized population. In addition to contributing to the body of knowledge on public health and nutrition in contexts of poverty, the findings can serve as a basis for the development of intersectional policies that promote equity and fair access to health services for informal waste pickers.

## 2. Materials and Methods

### 2.1. Study Design and Data Collection

Non-experimental, cross-sectional, and correlational design. The population included recyclers registered with local governments in three cities in Ecuador, Cuenca, La Libertad, and Macas, in 2023. Information was collected through a structured questionnaire that recorded sociodemographic data, working conditions, health status, experiences of workplace violence, and perceptions of gender stereotypes. Additionally, anthropometric measurements (weight, height, and waist circumference) and blood pressure were recorded, and laboratory tests (including stool, blood, and urine analyses) were conducted.

### 2.2. Variables

The dependent variable was nutritional status, operationalized using complementary anthropometric and biochemical indicators, in accordance with the ABCD framework of nutritional assessment. Given that the database included anthropometric and biochemical information, these two components were used to operationalize nutritional status. BMI was independently classified as normal weight or excess weight, and total lipid levels were categorized as normal or elevated based on previously established criteria.

For analytical purposes, both indicators were considered jointly in order to define comparison groups reflecting the presence of altered nutritional indicators. A value of 0 was assigned when both BMI and lipid levels were within normal ranges, whereas a value of 1 indicated an alteration in at least one of the two indicators (BMI and/or lipids). This dichotomous variable (0 = absence of nutritional alterations; 1 = presence of nutritional alterations) was constructed solely as an analytical tool and does not represent a composite clinical outcome.

A total of 34 sociodemographic, health, and work-related factors were considered as independent variables, including: sex; age; marital status; level of education; ethnic self-identification; presence of disability; receipt of financial assistance; leisure time; household size and income; hours spent on domestic work; city of collection; perception of health status; access to social security; medical consultations in the last twelve months; systolic and diastolic blood pressure; income from recycling; access to drinking water and public toilets during the working day; accidents at work; years in recycling; working hours; ability to ride a bicycle or tricycle and means of transportation to collection routes (on foot, bicycle, tricycle, bus, van, private car, or motorcycle).

### 2.3. Data Preprocessing

Missing values were imputed using the k-Nearest Neighbors (KNN) algorithm, which estimates data using the similarity between nearby observations [22]. Outliers were replaced by the median of each variable, a robust and appropriate measure for non-normal distributions. To incorporate categorical variables with more than two levels, One-Hot Encoding was applied, transforming each category into independent binary variables. The literature recommends this decision method, as tree-based algorithms improve predictive performance and facilitate the interpretation of results [23]. Before applying the classification models, a descriptive analysis of sociodemographic, occupational, and health characteristics was performed, segmented by nutritional status. Relative frequencies were calculated for categorical variables, and medians along with their respective interquartile ranges (IQRs) were reported for quantitative variables. Associations with nutritional status were evaluated using the Chi-square test (categorical) and the Mann–Whitney U test (quantitative), with *p*-values reported.

### 2.4. Machine Learning Analysis

The identification of the determinants of nutritional status was performed using supervised machine learning techniques based on decision trees: Random Forest (RF), Categorical Boosting (CatBoost or CB), and Extreme Gradient Boosting (XGBoost or XGB). These algorithms were chosen for their effectiveness in classification tasks, their ability to handle heterogeneous variables, and their capacity to evaluate the importance of each predictor [22,24,25]. RF combines multiple trees, improving accuracy and reducing overfitting. CB efficiently handles categorical variables and improves classification accuracy. XGB optimizes speed and employs advanced regularization to control overfitting.

The dataset was randomly divided into training (80%) and testing (20%) subsets. In an initial stage, baseline versions of each model were trained without applying explicit class imbalance correction techniques in order to establish a reference level of performance. Model performance was evaluated using overall accuracy, class-specific recall for Classes 0 and 1, and Receiver Operating Characteristic (ROC) analysis. Although accuracy was reported for completeness, it was not considered a reliable metric for model comparison given the pronounced class imbalance. Instead, class-specific recall and ROC-AUC were emphasized as more appropriate indicators of performance in this context.

ROC analysis was incorporated to provide a more robust and threshold-independent evaluation of the classifiers in light of the observed class imbalance [26]. ROC curves were constructed using predicted probabilities on the test set, and the area under the ROC curve (ROC-AUC) was calculated to assess the overall discriminative ability of the models across different decision thresholds.

To mitigate class imbalance, two complementary strategies were implemented. First, a data-level resampling approach using the Synthetic Minority Over-sampling Technique (SMOTE) was applied exclusively to the training set and embedded within the training process to prevent information leakage. Following oversampling, the models were retrained and evaluated using the same metrics applied to the baseline models. Second, a cost-sensitive learning strategy was employed through class-weighted training. For Random Forest, balanced class weights were applied. In the case of CatBoost, its native automatic class weighting mechanism (auto_class_weights = “Balanced”) was used. For XGBoost, class imbalance was addressed by incorporating balanced sample weights during model fitting. The performance of class-weighted models was evaluated using the same evaluation framework as that applied to the baseline and SMOTE-trained models, allowing for direct and consistent comparison across modeling strategies.

In addition, the relative importance of predictor variables was examined only for the model that achieved the highest ROC-AUC and exhibited the most balanced performance between classes, defined as a combination of superior discriminative ability and comparatively improved sensitivity toward the minority class. Feature importance plots display all predictors ranked in descending order according to their relative contribution to the classification task. A dashed reference line was included to highlight the ten most influential variables for visual interpretation purposes, while retaining the full set of predictors in the analysis.

To further support the interpretation of the most relevant predictors, heatmaps of class-wise mean differences were constructed using only the ten most influential variables identified by the selected model. These heatmaps provide a descriptive visualization of how key characteristics differ between Class 0 and Class 1. For each variable, mean values were calculated separately for Class 0 and Class 1, and the difference between classes (Class 1 − Class 0) was computed. For numerical variables, this difference reflects both the magnitude and direction of the contrast between classes. For binary categorical variables, such as sex (coded as 0 = male and 1 = female), the mean represents the proportion of individuals belonging to category 1. A diverging color scale centered at zero was used to facilitate a clear and rapid identification of contrasts between groups. These visualizations are descriptive in nature and do not fully explain the model’s predictive behavior, which arises from complex multivariate interactions.

All analyses were performed in the Google Colab environment using specialized Python libraries (version 3.12.12).

### 2.5. Ethical Approval

This study received ethical approval from the Comité de Ética de Investigación en Seres Humanos (CEISH) of the Universidad de Cuenca, Ecuador (Approval Code: 2023-003E0-VIUC). All participants provided written informed consent.

### 2.6. Data Availability

The dataset consists of fully anonymized records, including a hashed identification code. Due to the sensitive nature of the information and the vulnerable characteristics of the population, access to the dataset is restricted. Researchers may request access from the corresponding author and must sign a confidentiality and data-use agreement. De-identified aggregated data and Python code used for preprocessing and modeling are available upon reasonable request.

### 2.7. Use of Generative AI

Generative artificial intelligence tools were used exclusively for language editing during manuscript preparation and were not involved in study design, data processing, analysis, modeling, or interpretation.

## 3. Results

The study initially included 327 waste pickers; however, the final analysis was performed with 303 participants after excluding 24 cases due to insufficient data. The results are presented in three main sections.

### 3.1. Sociodemographic Characteristics, Working Conditions, and Health According to Nutritional Status

Table 1 shows sociodemographic, occupational, and health differences according to nutritional status. It can be seen that 29% (n = 87) of the sample corresponded to individuals without nutritional alterations (Class 0), and 71% (n = 216) presented nutritional alterations (Class 1), defined by the presence of excess body weight and/or altered lipid levels.

Regarding sociodemographic characteristics, a higher proportion of women was observed in Class 1 (76.4%) compared to Class 0 (54.0%) (*p* < 0.001), indicating a significant association with sex. Likewise, recyclers from Cuenca were more frequent in Class 1 (75.9%) (*p* = 0.022), while Class 0 predominated in La Libertad (27.6%) (*p* = 0.035). In addition, not knowing how to ride a bicycle was more common in Class 1 (72.2%), a significant difference (*p* = 0.048).

Age also showed differences, with a median age of 58 years in Class 0 and 53 years in Class 1 (*p* = 0.016). Overall, a higher likelihood of nutritional alterations was associated with being female, residing in Cuenca, and not using a bicycle. In contrast, living in La Libertad and older age were associated with a lower likelihood of nutritional alterations.

Table 2 summarizes the analysis of working conditions. Among the variables examined, only the use of bicycles as a means of transportation to the recycling route showed a significant association with nutrition alterations. The proportion of recyclers who use bicycles was higher in Class 0 than in Class 1 (9.2% versus 1.9%), suggesting that active transportation may be associated with a lower likelihood of nutritional alterations.

Table 3 presents the comparison of health-related characteristics. Only one variable showed significant differences between classes: attendance at medical check-ups during the previous 12 months. The proportion of recyclers who reported attending medical check-ups was significantly higher among individuals with nutritional alterations (44.4%) compared to those without nutritional alterations (29.9%) (*p* = 0.027). This finding suggests an association between healthcare utilization and the presence of nutritional alterations, possibly reflecting the need for clinical follow-up when such alterations are detected.

### 3.2. Performance of Classification Models

In this study, three classification algorithms based on decision trees were evaluated: RF, CB, and XGB. Table 4 summarizes the performance of these models under different class imbalance mitigation strategies, including baseline training, data-level resampling using SMOTE, and algorithm-level class weighting.

Overall accuracy values ranged from 0.67 to 0.74 across the different models and strategies. The highest accuracy was observed for the Random Forest model trained with class weighting. However, due to the pronounced class imbalance present in the dataset, accuracy was not considered a sufficient or reliable indicator of model performance and was therefore not used as a principal criterion for model selection.

When examining class-specific recall, a consistent pattern emerged across all models: performance was substantially better for Class 1 (individuals with nutritional alterations) than for Class 0 (individuals without nutritional alterations). Recall values for Class 1 were generally high, indicating a strong capacity of the models to identify individuals with nutritional alterations. In contrast, recall for Class 0 remained low in all cases, even after applying imbalance mitigation strategies, reflecting a limited ability to correctly identify individuals without nutritional alterations.

ROC-AUC values ranged from 0.51 to 0.62, indicating limited to moderate discriminative capacity among the evaluated models. The highest ROC-AUC value was achieved by the CatBoost model trained with SMOTE (0.62). Although this improvement was modest, this model also exhibited the most balanced performance between classes, combining higher overall discriminative ability with comparatively greater relative sensitivity toward the minority class.

Overall, the results indicate that even when class imbalance mitigation strategies are applied, a substantial improvement in the identification of individuals without nutritional alterations is not achieved. In this context, CatBoost with SMOTE was selected as the most informative model because it simultaneously maximized overall discriminative capacity (ROC-AUC) and showed greater relative sensitivity toward the minority class compared to the other models. While absolute performance for this class remained limited, the evaluated models demonstrated a high capacity to identify individuals with nutritional alterations. From a public health perspective, this is particularly relevant, as it supports the prioritization of interventions toward groups at higher nutritional risk rather than focusing on those without nutritional alterations.

### 3.3. Feature Importance Analysis (CatBoost Model Trained with SMOTE)

**Figure 1:**
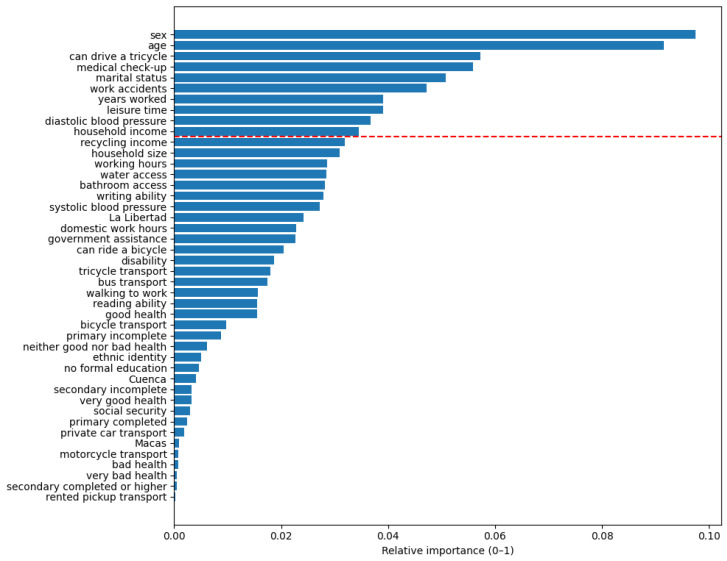
Variable importance in the CB model trained with SMOTE. **Note.** The figure presents the relative importance of variables estimated by the CatBoost model trained with SMOTE. Feature importance reflects each predictor’s contribution to the classification process within a multivariate framework and does not imply direct causal relationships with the presence of nutritional alterations. The dashed red line indicates the threshold separating the ten most influential variables.

Figure 1 presents the relative importance of predictor variables estimated by the CatBoost model trained with SMOTE, which achieved the highest ROC-AUC value and the most balanced performance between classes. The ten most influential variables together accounted for approximately 55% of the total model importance, suggesting that a relatively small set of features explains a substantial proportion of the model’s predictive capacity.

Among the variables with the greatest contribution to the model were sex and age, followed by other sociodemographic factors such as mobility ability (knowing how to drive a tricycle), marital status, leisure time, and household income. Variables related to working conditions, including years of employment and the occurrence of workplace accidents, also contributed to the classification process. Finally, variables related to health and access to healthcare, such as attendance at medical check-ups and diastolic blood pressure, were identified as relevant predictors.

These results indicate that the presence of nutritional alterations among waste pickers is associated with a combination of sociodemographic, occupational, economic, and health-related factors rather than a single isolated determinant. It is important to emphasize that feature importance reflects each variable’s contribution to the classification process within a multivariate and non-linear framework and does not imply direct causal relationships, but rather complex patterns that differentiate individuals with and without nutritional alterations.

####  Visual Comparison of Class Differences for the Most Relevant Features (CatBoost Model Trained with SMOTE)

**Figure 2:**
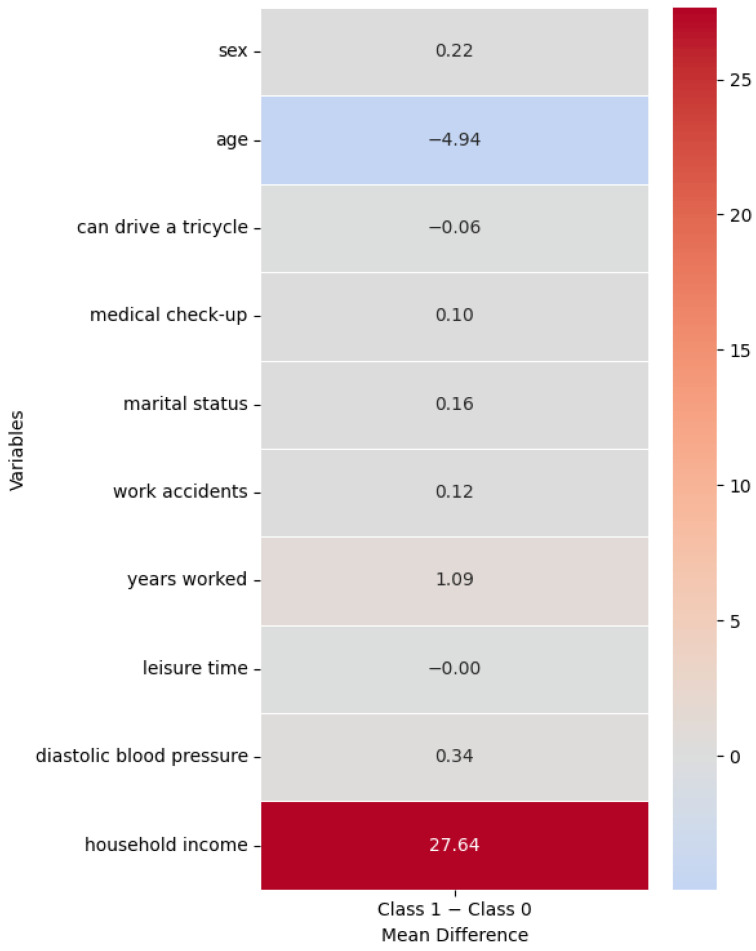
Heatmap of mean differences between classes. **Note.** The figure shows the mean differences between Class 1 (individuals with nutritional alterations) and Class 0 (individuals without nutritional alterations). Values close to zero indicate low descriptive differentiation between groups. These differences are descriptive in nature and do not necessarily reflect the predictive importance of variables in the machine learning models.

Figure 2 presents a heatmap of mean differences between Class 1 (recyclers with nutritional alterations) and Class 0 (recyclers without nutritional alterations) for the ten most relevant variables identified by the CatBoost model trained with SMOTE.

Several contrasts between classes can be observed. The most pronounced difference corresponds to household income, with Class 1 exhibiting substantially higher values than Class 0. On average, households of recyclers with nutritional alterations reported incomes approximately USD 27.64 higher than those of recyclers without nutritional alterations. This finding suggests that, within this specific population, higher household income does not necessarily translate into a lower likelihood of nutritional alterations.

Another notable difference was observed for age, as individuals in Class 1 were, on average, 4.94 years younger than those in Class 0. This indicates a higher prevalence of nutritional alterations among younger recyclers in the analyzed sample, a pattern that differs from traditional associations reported in the literature and highlights the importance of contextual and occupational factors in shaping nutritional risk.

For the variable sex (coded as 0 = male and 1 = female), the positive mean difference indicates a higher proportion of women in Class 1 compared to Class 0. The remaining variables showed smaller differences, in some cases close to zero, suggesting less pronounced contrasts between groups. Nevertheless, factors related to working conditions (years of work and workplace accidents), access to healthcare (medical check-ups), and clinical indicators such as diastolic blood pressure contributed in a complementary manner to class differentiation within the model.

Overall, the heatmap indicates that individuals with nutritional alterations are primarily characterized by higher household income, younger age, and a greater proportion of women compared to those without nutritional alterations. These descriptive contrasts help contextualize the feature importance results but do not fully explain the model’s predictive behavior, which reflects complex multivariate interactions rather than isolated or purely descriptive differences between groups.

## 4. Discussion

The sociodemographic, occupational, and health characteristics of the waste pickers analyzed are consistent with previous literature, which describes this population as highly exposed to precarious working conditions, low income, limited access to basic services during the workday, and scarce educational opportunities [6,14,15,16,17,27]. From a public health perspective, the fact that 71% of the studied population presents nutritional deficiencies highlights the magnitude of the problem in this occupational group. This finding agrees with previous studies that report a high prevalence of overnutrition among waste pickers and other socially vulnerable populations, such as women and older adults [12,28], suggesting the persistence of this phenomenon in contexts of structural disadvantage.

In this study, machine learning techniques were used to identify factors associated with the presence of nutritional deficiencies in waste pickers in Ecuador, using three decision tree-based algorithms: Random Forest (RF), CatBoost (CB), and XGBoost (XGB). Although overall accuracy values ranged from 0.67 to 0.74, this metric was not considered suitable as a primary performance criterion due to the significant class imbalance present in the data. Consequently, the evaluation focused on metrics more appropriate for this context, such as class-specific recall and ROC-AUC analysis, following methodological recommendations for unbalanced classification problems.

Despite the application of strategies to mitigate class imbalance, such as resampling using SMOTE and class-weighted training, the results show that no substantial improvement was achieved in the identification of the minority class (recyclers without nutritional deficiencies). In all models evaluated, recall was consistently higher for Class 1 (recyclers with nutritional deficiencies) than for Class 0 (recyclers without nutritional deficiencies). This pattern should not be interpreted as an intrinsic limitation of the algorithms, but rather as a consequence of the dataset structure and the high overall vulnerability of the studied population, in which individuals without nutritional deficiencies constitute a small group that is difficult to distinguish using supervised classification techniques.

Among the models evaluated, the CatBoost model trained with SMOTE achieved the highest ROC-AUC value (0.62) and showed the best relative balance between classes, although this improvement was modest in absolute terms. For this reason, this model was selected as the most informative for exploratory and interpretive analysis, particularly for identifying multivariate patterns associated with the presence of nutritional deficiencies. It is important to emphasize that none of the evaluated models are suitable as a clinical screening tool; however, their value lies in their ability to explore complex interactions among multiple factors in a highly vulnerable population, rather than in their individual predictive use.

The analysis of variable importance based on the CatBoost model trained with SMOTE identified a set of sociodemographic, occupational, economic, and health factors as the most relevant predictors. These included sex, age, mobility ability (knowing how to drive a tricycle), marital status, leisure time, and household income. Likewise, variables related to working conditions, such as years of employment and the occurrence of workplace accidents, along with health variables and access to healthcare services, such as attendance at medical check-ups and diastolic blood pressure, contributed significantly to the classification process. These variables should be interpreted within a multivariate and nonlinear framework, in which a predictor can be relevant even when the descriptive differences between groups are small.

Sex emerged as the most influential predictor, consistent with previous evidence documenting a higher prevalence of nutritional deficiencies among female recyclers [11,12,13]. However, the descriptive differences between classes were limited, suggesting that their predictive relevance is mediated by social and economic determinants, such as a heavier burden of domestic work, less access to resources, and persistent structural constraints that disproportionately affect women [11,28,29]. These factors reinforce their nutritional vulnerability in contexts of informal employment.

Age was the second most relevant predictor; however, unlike what has been reported in other contexts [13,28,30], waste pickers with nutritional deficiencies were, on average, younger than those without. This difference can be explained by the particularities of the analyzed context, where younger generations prioritize intensive work in precarious conditions, with diets of low nutritional quality, little time for physical activity, and limited access to health services. The available evidence on informal waste pickers, although limited in the analysis by age, describes a heterogeneous nutritional status influenced by job insecurity and food insecurity [12,31].

Likewise, the variable “knows how to drive a tricycle,” which can be interpreted as a proxy for physical activity, showed a significant contribution to the model, consistent with the literature that links the use of active transportation with better health conditions. Other variables, such as marital status, workplace accidents, years of employment, and leisure time, while not showing statistically significant associations in the descriptive analyses, contributed information to the model when integrated into more complex multivariate patterns. This highlights the ability of machine learning approaches to capture interactions that are undetectable through traditional univariate analyses.

Finally, household income, although not showing a direct statistical association with the presence of nutritional deficiencies, was one of the most influential predictors in the model. The fact that recyclers with nutritional deficiencies have slightly higher incomes than those without deficiencies contrasts with studies that associate malnutrition primarily with extreme poverty [12]. This apparent contradiction can be explained by the low variability of income in this population and by the predominance of food environments characterized by foods with high caloric density and low nutritional value, where higher incomes do not guarantee a healthier diet.

Taken together, the results confirm that nutritional status, operationalized in this study through the presence or absence of nutritional deficiencies, responds to a complex interaction of social, occupational, economic, and health factors, rather than to isolated determinants. From a public health perspective, the ability of these models to more accurately identify individuals with nutritional deficiencies is particularly relevant, as it allows for the design of policies and interventions targeting the groups at highest risk, while also underscoring the need for comprehensive strategies that address the structural causes of nutritional vulnerability in this population.

However, the results should be interpreted considering some methodological limitations. First, the cross-sectional design of the study prevents establishing causal relationships between the variables analyzed and the presence of nutritional alterations. Second, nutritional status was operationalized using the available anthropometric and biochemical indicators (BMI and lipid levels), with the aim of integrating the existing information and approximating the concept of nutritional alterations from a complementary perspective. In this context, it was not feasible to conduct sensitivity analyses evaluating BMI- and lipid-related alterations separately, both because the analytical objective focused on nutritional status operationalized as the presence of nutritional alterations and due to limitations in sample size. Nevertheless, conducting differentiated analyses is proposed for future research, particularly in studies with larger samples, considering that machine learning algorithms tend to perform more efficiently when larger volumes of data are available. Likewise, the marked class imbalance affected the performance of the machine learning models, limiting the identification of individuals without nutritional alterations despite the application of mitigation strategies. Finally, the models used had an exploratory and descriptive purpose, aimed at identifying multivariate patterns and supporting public health planning, and should not be interpreted as tools for individual clinical screening.

## 5. Conclusions

This study identified a high prevalence of nutritional deficiencies (71%) among grassroots recyclers, revealing a public health problem in a vulnerable working group and highlighting the need to recognize this population as a priority within public health and social protection agendas.

Nutritional deficiencies were significantly associated with sociodemographic, health, and territorial variables, such as sex, canton of residence, bicycle riding, bicycle use as a means of transportation to work, age, and regular medical check-ups, allowing the identification of differentiated risk profiles within this population. Higher proportions of nutritional deficiencies were observed in women, in people residing in the Cuenca canton, in those who do not know how to ride a bicycle, and in those who do not use a bicycle as a means of transportation to work. It was also found that those presenting these deficiencies had a lower average age, suggesting nutritional risks at earlier stages in this population and reinforcing the need to consider a life-cycle approach in intervention strategies. Additionally, it was found that the proportion of waste pickers who attended a medical check-up was significantly higher among those with nutritional deficiencies, indicating greater use of health services in this group and suggesting the possible presence of clinical symptoms. This underscores the need to strengthen prevention, promotion, early detection, follow-up, and timely treatment efforts.

The application of supervised machine learning models proved useful as a support tool for planning, prioritizing, and evaluating public health strategies, facilitating the identification of population subgroups with a higher probability of presenting nutritional deficiencies. While these models are not intended as clinical screening tools, they provide relevant evidence for a more efficient allocation of available resources in contexts of social vulnerability.

In conclusion, these results support and strengthen, with evidence, the design of public policies and targeted intervention strategies that incorporate periodic nutritional assessment and monitoring of the waste picker population. These strategies integrate approaches based on equity, territory, life cycle, and occupational health, with the aim of improving the health conditions and quality of life of this population.

## Figures and Tables

**Table 1:** Comparison of sociodemographic characteristics according to nutritional alterations (Class 0 vs. Class 1).

Variable	Category	Class 0 (%) n = 87	Class 1 (%) n = 216	Test Statistic	*p*-Value
Sex	Male	46.0	23.6	χ^2^ = 13.72	0.000 *
	Female	54.0	76.4		
Marital status	No partner	51.7	42.1	χ^2^ = 1.94	0.164
	With partner	48.3	57.9		
Can read	No	25.3	19.0	χ^2^ = 1.14	0.286
	Yes	74.7	81.0		
Can write	No	26.4	19.9	χ^2^ = 1.19	0.275
	Yes	73.6	80.1		
Self-identification	Other	11.5	9.7	χ^2^ = 0.64	0.887
	Mestizo	88.5	90.3		
Has a disability	No	78.2	81.9	χ^2^ = 0.36	0.551
	Yes	21.8	18.1		
Receives vouchers or assistance	No	74.7	69.4	χ^2^ = 0.60	0.439
	Yes	25.3	30.6		
Cuenca	No	37.9	24.1	χ^2^ = 5.23	0.022 *
	Yes	62.1	75.9		
La Libertad	No	72.4	83.8	χ^2^ = 4.42	0.035
	Yes	27.6	16.2		
Macas	No	89.7	92.1	χ^2^ = 0.22	0.639
	Yes	10.3	7.9		
No educational level	No	80.5	88.4	χ^2^ = 2.66	0.103
	Yes	19.5	11.6		
Incomplete primary education	No	70.1	68.1	χ^2^ = 0.05	0.832
	Yes	29.9	31.9		
Complete primary education	No	67.8	64.8	χ^2^ = 0.13	0.716
	Yes	32.2	35.2		
Incomplete secondary education	No	90.8	89.8	χ^2^ = 0.00	0.961
	Yes	9.2	10.2		
High school graduate and above	No	90.8	88.9	χ^2^ = 0.08	0.776
	Yes	9.2	11.1		
Leisure time	No	56.3	56.0	χ^2^ = 0.00	1.000
	Yes	43.7	44.0		
Rides a bicycle	No	59.8	72.2	χ^2^ = 3.91	0.048 *
	Yes	40.2	27.8		
Rides a tricycle	No	48.3	57.9	χ^2^ = 1.94	0.164
	Yes	51.7	42.1		
Continuous variables (Median [IQR], Test statistic, *p*-value)					
Age		58.0 [44.0–67.5]	53.0 [40.7–61.0]	U = 11,059.00	0.016 *
Total number of household members		4.0 [2.0–6.0]	4.0 [3.0–6.0]	U = 9755.00	0.600
Household income		180.0 [145.0–300.0]	200.0 [150.0–310.0]	U = 8621.50	0.261
Hours of domestic work		3.0 [2.0–6.0]	4.0 [2.0–5.0]	U = 8589.50	0.238

**Note:** Categorical variables were compared using the χ^2^ test, and quantitative variables were compared using the Mann–Whitney U test. Values are presented as percentages for categorical variables and as median [IQR] for quantitative variables. * *p* < 0.05.

**Table 2:** Comparison of working conditions according to nutritional alterations (Class 0 vs. Class 1).

Variable	Category	Class 0 (%) n = 87	Class 1 (%) n = 216	Statistic	*p*-Value
Access to bathrooms	No	67.8	68.5	χ^2^ = 0.00	1.000
	Yes	32.2	31.5		
Access to water	No	69.0	71.3	χ^2^ = 0.07	0.792
	Yes	31.0	28.7		
Workplace accidents	No	67.8	58.3	χ^2^ = 1.96	0.161
	Yes	32.2	41.7		
Walking	No	49.4	44.4	χ^2^ = 0.44	0.509
	Yes	50.6	55.6		
Bicycle transport	No	90.8	98.1	χ^2^ = 6.97	0.008 *
	Yes	9.2	1.9		
Tricycle transfer	No	71.3	78.7	χ^2^ = 1.52	0.217
	Yes	28.7	21.3		
Bus transfer	No	88.5	81.5	χ^2^ = 1.74	0.187
	Yes	11.5	18.5		
Van rental transfer	No	95.4	95.4	χ^2^ = 0.00	1.00
	Yes	4.6	4.6		
Private car transfer	No	93.1	92.1	χ^2^ = 0.00	0.960
	Yes	6.9	7.9		
Motorcycle transport	No	94.3	96.3	χ^2^ = 0.23	0.631
	Yes	5.7	3.7		
Continuous variables (Median [IQR], Test statistic, *p*-value)					
Recycling income		100.0 [70.0–145.0]	100.0 [80.0–150.0]	U = 9028.00	0.592
Working hours		6.0 [4.0–8.0]	6.0 [4.0–8.0]	U = 9197.50	0.772
Years of work		13.0 [6.0–21.0]	15.0 [8.0–20.0]	U = 8598.00	0.247

* *p* < 0.05.

**Table 3:** Comparison of health conditions according to nutritional alterations (Class 0 vs. Class 1).

Variable	Category	Class 0 (%) n = 87	Class 1 (%) n = 216	Statistic	*p*-Value
Perception of health is very poor	No	97.7	94	χ^2^ = 1.12	0.290
	Yes	2.3	6		
Poor health perception	No	92	90.3	χ^2^ = 0.06	0.813
	Yes	8	9.7		
Perception of health is neither good nor bad	No	48.3	44.9	χ^2^ = 0.16	0.686
	Yes	51.7	55.1		
Perception of good health	No	69	73.6	χ^2^ = 0.46	0.499
	Yes	31	26.4		
Very good health perception	No	93.1	97.2	χ^2^ = 1.79	0.181
	Yes	6.9	2.8		
Consultation control	No	70.1	55.6	χ^2^ = 4.88	0.027 *
	Yes	29.9	44.4		
Social security	No	90.8	91.2	χ^2^ = 0.00	1.000
	Yes	9.2	8.8		
Continuous variables (Median [IQR], Test statistic, *p*-value)					
Systolic		110.0 [105.0–125.0]	114.0 [105.0–121.0]	U = 9094.00	0.660
Diastolic		71.0 [70.0–80.0]	71.0 [70.0–80.0]	U = 8744.50	0.337

* *p* < 0.05.

**Table 4:** Performance of machine learning models under different class imbalance mitigation strategies.

Model	Strategy	Accuracy	Recall		ROC-AUC
			Class 0	Class 1	
RF	Baseline	0.72	0.12	0.96	0.51
RF	SMOTE	0.62	0.12	0.82	0.53
RF	Class-weighted	0.74	0.06	1.00	0.51
CB	Baseline	0.71	0.06	0.96	0.54
CB	SMOTE	0.64	0.18	0.82	0.62
CB	Class-weighted	0.66	0.18	0.84	0.58
XGB	Baseline	0.66	0.18	0.84	0.53
XGB	SMOTE	0.66	0.18	0.84	0.53
XGB	Class-weighted	0.69	0.18	0.89	0.56

## Data Availability

The data presented in this study are not publicly available due to ethical and privacy restrictions related to the sensitive nature of the information and the vulnerability of the study population. The dataset consists of fully anonymized records with hashed identification codes. De-identified aggregated data and the Python code used for data preprocessing and modeling are available from the corresponding author upon reasonable request and subject to the signing of a confidentiality and data-use agreement.

## Abbreviations

The following abbreviations are used in this manuscript:

BMI	Body Mass Index
KNN	K-Nearest Neighbors
IQR	Interquartile range
RF	Random Forest
CB	Categorical Boosting
XGB	Extreme Gradient Boosting
CEISH	Comité de Ética en Seres Humanos
